# Chemosensory deficits are best predictor of serologic response among individuals infected with SARS-CoV-2

**DOI:** 10.1371/journal.pone.0274611

**Published:** 2022-12-14

**Authors:** Jonathan B. Overdevest, Alexandria L. Irace, Valeria Mazzanti, Eun Jeong Oh, Paule V. Joseph, Davangere P. Devanand, Zachary C. Bitan, Eldad A. Hod, David A. Gudis, Codruta Chiuzan

**Affiliations:** 1 Department of Otolaryngology-Head and Neck Surgery, Columbia University Irving Medical Center, NewYork-Presbyterian Hospital, New York, NY, United States of America; 2 Department of Biostatistics, Columbia University, New York, NY, United States of America; 3 Department of Biostatistics, Epidemiology and Informatics, University of Pennsylvania, Philadelphia, PA, United States of America; 4 National Institute of Alcohol Abuse and Alcoholism, Section of Sensory Science and Metabolism & National Institute of Nursing Research, Bethesda, MD, United States of America; 5 Department of Psychiatry, Columbia University Irving Medical Center, New York, NY, United States of America; 6 Division of Geriatric Psychiatry, New York State Psychiatric Institute, New York, NY, United States of America; 7 Department of Pathology and Cell Biology, Columbia University, New York, NY, United States of America; 8 Institute of Health System Science Feinstein Institutes for Medical Research Northwell Health, New York, NY, United States of America; Kaohsuing Medical University Hospital, TAIWAN

## Abstract

**Objective:**

Smell and taste alteration are closely linked to infection with SARS-CoV-2 and may be associated with a more indolent disease course. Serologic response rates among individuals with mild disease remains limited. We sought to identify whether chemosensory changes associated with COVID-19 were predictive of a serologic response.

**Study design:**

Cross-sectional study.

**Methods:**

The sample consisted of 306 adults (≥18 years old) volunteering for convalescent plasma donation following perceived COVID-19 illness from April-June 2020. Documentation of COVID-19 PCR status, clinical symptoms at time of illness, and treatment course occurred at the time of serologic analysis, where we assessed chemosensory function using patient-perceived deficits. We implemented previously validated ELISA screening to determine serologic status regarding anti-Spike immunoglobulins. Statistical analysis using stepwise logistic models were employed to identify predictive factors of serologic response.

**Results:**

Of 306 patients undergoing serologic and chemosensory evaluation, 196 (64.1%) and 195 (63.7%) reported subjective olfactory and taste dysfunction, respectively, during the first two weeks of COVID-19 infection. In unadjusted models, the odds of developing suprathreshold IgG antibody titers were 1.98 times higher among those who reported altered smell (95% CI 1.14–3.42, p = 0.014) and 2.02 times higher among those with altered taste (95% CI 1.17–3.48, p = 0.011) compared to those with normal smell and taste. Multivariable logistic models adjusting for sex, age, race/ethnicity, symptom duration, smoking status and comorbidities index demonstrated that altered smell and taste remained significant predictors of positive anti-spike IgG response (smell OR = 1.90, 95% CI 1.05–3.44, p = 0.033; taste OR = 2.01, 95% CI = 1.12–3.61, p = 0.019).

**Conclusion:**

Subjective chemosensory dysfunction, as self-reported smell or taste deficiency, is highly predictive of serologic response following SARS-CoV-2 infection. This information may be useful for patient counseling. Additional longitudinal research should be performed to better understand the onset and duration of the serologic response in these patients.

## Introduction

As the coronavirus disease 2019 (COVID-19) pandemic unfolded, chemosensory dysfunction, or impairment of smell and/or taste, was identified as a key symptom of severe acute respiratory syndrome coronavirus (SARS-CoV-2) infection [1, 2]. Recent meta-analyses report that olfactory loss is present in 43% to 62% of COVID-19 patients [3], though this estimate may vary based on ethnicity, age, disease severity, and method of chemosensory evaluation [4–7]. In addition to its high prevalence, olfactory loss typically presents early in the course of the disease, making it a useful sentinel symptom to prompt further testing or management [8]. Approximately 20% of patients may have no symptoms other than a diminished sense of smell, and there may be an association between chemosensory dysfunction and milder disease severity [2, 6, 9–12]. As the magnitude of immunological response to SARS-CoV-2 may be associated with the severity of disease [13], we sought to explore the link between serologic response and chemosensory loss.

The pathophysiological mechanism underlying chemosensory dysfunction remains under investigation. Transient olfactory loss concomitant with endemic viral infections is thought to be related to inflammatory changes in the nasal cavity that prevent odorants from reaching the olfactory epithelium [14, 15]. SARS-CoV-2, however, displays tropism for the angiotensin-converting enzyme 2 (ACE2) receptor expressed on supporting sustentacular and horizontal basal cells within the olfactory epithelium. Viral entry into these supporting cells in turn may cause cellular damage and transient disruption of the integrity of the ciliated olfactory epithelium [16]. Recent evidence suggests that SARS-CoV-2 infection impairs olfaction by disrupting the nuclear architecture of olfactory sensory neurons, in turn resulting in loss of olfactory receptor expression required for smell perception [17].

Understanding the natural immune response to SARS-CoV-2 can help elucidate the disease pathophysiology, recognize epidemiological patterns, and guide interventions. Immunocompetent individuals typically mount an adaptive immune response to SARS-CoV-2, during which antibodies are produced against specific viral antigens: the nucleocapsid (N) protein and spike (S) protein. The spike protein includes the S1 and S2 subunits, and the S1 subunit contains the receptor binding domain (RBD) which is not only the main mediator of viral entry to human cells, but also the key target for neutralizing antibodies. IgM, IgG, and IgA against the spike protein subunits can be detected 1–3 weeks after initial infection, but the duration of seropositivity is unknown. It has been reported that antibodies may confer protection from COVID-19 reinfection for at least 6 months [18], although these protective antibodies do not guarantee immunity [19], particularly for phylogenetically distinct strains [20]. Moreover, there is a reported direct relationship between disease severity and increased antibody titers [21–23]. Rates of reinfection and seroreversion have been found to be higher among individuals with mild or asymptomatic primary infection, who may be more likely to be seronegative [24]. Although recent analyses report guarded prognostic value for olfactory function in predicting disease severity [25], other studies suggest a trend toward milder disease among individuals experiencing chemosensory dysfunction [2, 26], who in turn may be at risk for diminished serologic response and possible reinfection. For example, one meta-analysis demonstrated that self-reported altered smell was significantly more prevalent among those with mild-to-moderate COVID-19 symptoms than severe symptoms (67% vs 31%, p<0.05) [6]. Separate retrospective studies also demonstrated that smell loss was significantly more common in non-hospitalized patients (70.1%) than hospitalized patients (48.7%) [26], and patients reporting olfactory dysfunction as symptoms of COVID-19 were ten times less likely to be admitted to the hospital than those with normal sense of smell (OR 0.09, 95% CI 0.01–0.74) [10].

Acute smell loss has been identified as a strong predictor of COVID-19 [27]; however, little is known about how chemosensory dysfunction relates to humoral response and immunity to SARS-CoV-2. One previous study of a sample based in Paris identified a higher percentage of seropositivity among those with chemosensory dysfunction [28]. The current study expands upon these findings using multivariable prediction models with a sample from a large, diverse urban center in the United States. The objective of this study is to characterize the development of anti-Spike protein immunoglobulins among COVID-19 patients with smell and taste loss to explore which clinical symptoms are most predictive of a robust serologic response.

## Methods

### Study participants

**Fig 1:**
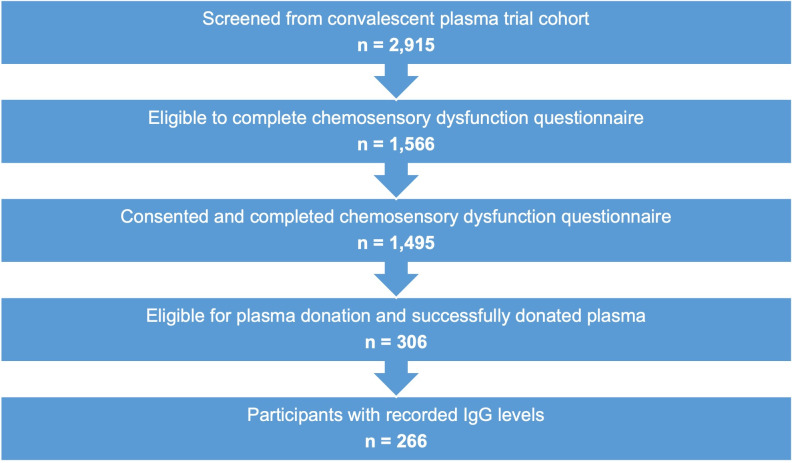
Flow diagram illustrating participant selection.

Participants were adults (≥18 years of age) enrolled in the NewYork-Presbyterian/Columbia University Irving Medical Center convalescent plasma trials (CPT) after a history of recent illness consistent with COVID-19 (Fig 1). Eligibility for CPT has been described previously [29]. Eligible participants had evidence of active or previous SARS-CoV-2 infection by: PCR from nasopharyngeal swabs or antibody-positive serology within fourteen days of randomization, or other clinical signs of infection on chest x-ray, pulse oximetry, or by respiratory/ventilatory requirements. Eligible participants also tested negative for transfusion-transmitted infections (e.g. human immunodeficiency virus (HIV), hepatitis B & C viruses (HBV/HCV), west nile virus (WNV), human T-lymphotropic virus type I & II (HTLV-I/II), *Trypanosoma cruzi*, and Zika virus). Participants were excluded if they had received an anti-viral agent within 24 hours of plasma donation; required mechanical ventilation or extracorporeal membrane oxygenation for ≥5 days; were diagnosed with severe multi-organ failure, IgA deficiency, pregnancy; or had a history of allergic transfusion reactions. Participants largely drew from the Northern Manhattan geographic region.

Participants included in this study passed eligibility screening to participate in convalescent plasma donation, had evidence of previous COVID-19, had a minimum of two weeks following resolution of acute-phase symptoms and demonstrated no signs of active infection at the time of donation. Other than persistence of chemosensory dysfunction, long-haul or post-acute sequelae of COVID (PASC) symptoms were not assessed at the time of enrollment. Study participants underwent standard apheresis blood collection at the New York Blood Center between April 2020 through June 2020, following the initial wave of infections in New York, NY. Consent was obtained from these participants to complete additional study instruments exploring chemosensory issues associated with COVID-19, as described below. This study protocol (AAAT0264) was approved by the Institutional Review Board at Columbia University Irving Medical Center.

### Questionnaire description and data extraction

Participants completed an additional survey evaluating 1) subjective smell and taste function at baseline and during COVID-19 (S1 Table) to identify pre-morbid anosmic individuals, 2) symptoms included in the rhinologic subdomain of the Sino-nasal Outcome Test-22 (SNOT-22), 3) history of COVID-related symptoms, and 4) COVID testing history. Additional data collected included demographic variables, smoking status, and comorbidities (heart disease, hypertension, lung disease, diabetes mellitus, stomach issues, kidney disease, liver disease, hematologic disease, cancer, depression, osteoarthritis, back pain, rheumatoid arthritis, and other medical issues).

### Elisa antibody testing

Enzyme-linked immunosorbent assay (ELISA) was used to quantitate anti-spike SARS-CoV-2 antibody titers for each cohort. Antibodies were characterized based on IgM and IgG immunoglobulin classes representing primary and durable immune responses, respectively, following previously described protocols [30]. Briefly, 96-well ELISA plates were coated with the SARS-CoV-2 spike trimer and N at 4°C overnight. The plates were washed using phosphate buffered saline (PBS) and blocked using PBS with 3% nonfat dry milk. Serial dilutions of plasma samples were applied to each well. Dilutions were prepared using PBS with 0.1% Tween 20 + 10% fetal calf serum. Sample dilutions began at 1:100 and were successively diluted fourfold five times. Plates were incubated for 1 hour at 37°C. After washing, peroxidase AffiniPure goat anti-human IgG (H+L) antibody (1:3,000 dilution), anti-human IgA antibody (1:5,000 dilution, Jackson ImmunoResearch), or anti-human IgA antibody (1:5,000 dilution, Thermo Fischer Scientific) was added to each well, followed by another hour of incubation at 37°C. Plates were washed and the reaction was stopped using 1M sulfuric acid. Absorbance was quantified as optical density (OD) absorbed at 450 nm wavelength. Convalescent plasma was screened as positive for study inclusion in cases of a minimum anti–SARS-CoV-2 total IgG antibody titer of at least 1:400, as evaluated by quantitative ELISA against the spike protein.

### Statistical analysis

Descriptive statistics were summarized as frequencies (percentages) for the categorical variables and median (inter-quartile range IQR) for age (continuous variable). Based on the clinical relevance and frequency distributions, age was categorized into “18–30", “31–50” and “51+”. Participant’s race/ethnicity was dichotomized into “White/Non-Hispanic” or “Other”. The primary outcome for our regression models was status as “IgG positive” (>0.215 optical density [OD], a measure of absorbance) or “IgG negative” (≤0.215 OD), the threshold seropositivity criteria for CPT. We employed both univariable and multivariable logistic regressions to model the association between IgG positivity and age, race, sex, duration of symptoms, hospitalization, presence of fever, smoking status, comorbidity burden, and alterations in smell or taste. All variables tested in univariable analyses were entered into a multivariable model with stepwise method based on the Akaike Information Criterion (AIC) criterion (where smaller is better), which was used to select variables that provide the best fit for the model. The performance, or discriminative ability of the models, was assessed using the area under the ROC curve (AUC), area under the precision-recall curve (AUPRC), and calibration slope. All statistical analyses were performed in R (v. 4.1.0) using a two-sided type I error of 0.05.

## Results

### Study population

Data on smell and taste were available for 306 participants meeting inclusion criteria for CPT and having complete consent records to be included in this study. Baseline characteristics of the full study cohort are displayed in Table 1. Median participant age was 39.0 years (IQR 32.0–50.0). Most participants in the CPT cohort were female (n = 196, 64.1%) and white (n = 262, 85.6%). Self-report of pre-morbid baseline smell and taste were subjectively normal in most participants (smell: n = 294, 96.4%; taste: n = 300, 98.7%), where participants identified as lacking smell or taste awareness consistent with pre-existing anosmia or ageusia were excluded. Smell, taste, and concomitant chemosensory alteration (smell and taste) during the first two weeks COVID-19 illness were noted by 196 participants (64.1%), 195 participants (63.7%), and 177 (57.8%) participants, respectively. Among those reporting smell alteration, 9 participants had more than one answer to questions characterizing the quality of smell dysfunction and were disregarded. The remaining 187 valid answers revealed 135 (72.2%) reporting absent perception of smell during their illness, 37 (19.8%) with diminished smell, 3 (1.6%) heightened smell, 10 (5.3%) distorted smell, and 2 (1.1%) odd smell.

**Table 1:** Baseline characteristics of the study cohort (n = 306).

Characteristic (total N with available data)	N (%)
**Sex** (female)	196 (64.1)
**Age** (years; median [IQR])	39 (32.0–50.0)
**Race/Ethnicity**	
White (non-Hispanic)	262 (85.6)
Asian	14 (4.6)
Black	2 (0.7)
Native American/Pacific Islander	1 (0.3)
Other	27 (8.8)
**Smoking** (n = 304)	
Current smoker	5 (1.6)
Former smoker	26 (8.6)
Non-smoker	273 (89.8)
**Hospitalized for COVID-19** (n = 302)	5 (1.7)
**Symptom duration** (days, n = 304)	
0–3	22 (7.2)
4–7	60 (19.7)
8–11	65 (21.4)
12–15	92 (30.3)
>15	65 (21.4)
**Other presenting symptoms**	
Fever (n = 292)	162 (55.5)
Cough (n = 287)	144 (50.2)
Dyspnea (n = 275)	85 (30.9)
**Smell alteration** (n = 306)	
Normal	101 (33.0)
Altered	196 (64.1)
Unanswered/Other	9 (2.9)
**Taste alteration** (n = 306)	
Normal	102 (33.3)
Altered	195 (63.7)
Unanswered/Other	9 (2.9)
**Medical Comorbidities** (n = 305)	
0	185 (60.7)
1	19 (6.2)
≥ 2	101 (33.1)

### Associations between clinical characteristics and serologic response

Out of a total of 306 participants, 40 (13.1%) had missing IgG values. Of the 266 remaining individuals with complete IgG data, initial antibody screening demonstrated detectable levels of SARS-CoV-2 anti-Spike IgG in 176 individuals (66.2%), while 90 individuals (33.8%) were deemed antibody negative based on a cut-off of 0.215 OD (Table 2), a standardized threshold used for participation in CPT donation. There were significantly more female participants who were SARS-CoV-2 antibody negative (78.9%) than positive (58.0%, p<0.001). Distribution of other demographic characteristics, including age and race/ethnicity, did not differ based on serologic response.

**Table 2:** Baseline characteristics by SARS-CoV-2 antibody response (n = 266).

Characteristic	SARS-CoV-2 antibody positive (n = 176)	SARS-CoV-2 antibody negative (n = 90)	p-value**
**Sex** (female)	102 (58.0%)	71 (78.9%)	**< .001**
**Age** (years; median [IQR])	40 (32–51)	39 (33.2–50)	0.844
**Race/Ethnicity**			0.345
White (non-Hispanic)	148 (84.1%)	83 (92.2%)	
Asian	10 (5.7%)	3 (3.3%)	
Black	2 (1.1%)	0 (0%)	
Native American/Pacific Islander	0 (0%)	0 (0%)	
Other	16 (9.1%)	4 (4.4%)	
**Smoking**			0.782
Current smoker	3 (1.7%)	1 (1.1%)	
Former smoker	14 (8.0%)	9 (10.2%)	
Non-smoker	159 (90.3%)	78 (88.6%)	
**Hospitalization** (yes)	3 (1.7%)	1 (1.1%)	0.703
**Symptom duration** (days)			0.573
0–3	9 (5.1%)	9 (10.1%)	
4–7	34 (19.4%)	16 (18.0%)	
8–11	41 (23.4%)	18 (20.2%)	
12–15	50 (28.6%)	28 (31.5%)	
>15	41 (23.4%)	18 (20.2%)	
**Other presenting symptoms**			
Fever	92 (55.1%)	46 (53.5%)	0.809
Cough	79 (48.8%)	44 (51.2%)	0.719
Dyspnea	38 (24.5%)	33 (40.2%)	**0.012**
Smell alteration			**0.047**
Normal	45 (25.6%)	36 (40.0%)	
Altered	126 (71.6%)	51 (56.7%)	
Unanswered/Other	5 (2.8%)	3 (3.3%)	
Taste alteration			**0.033**
Normal	45 (25.6%)	37 (41.1%)	
Altered	125 (71.0%)	51 (56.7%)	
Unanswered/Other	6 (3.4%)	2 (2.2%)	
SNOT-22 rhinologic subdomains*, ≥ 3 points (moderate/severe)			
Need to blow nose	13 (7.3%)	11 (12.4%)	0.280
Nasal Blockage	16 (9.1%)	10 (11.3%)	0.614
Sneezing	6 (3.4%)	6 (6.7%)	0.282
Runny Nose	7 (4.0%)	3 (3.3%)	0.304
Cough	7 (4.0%)	5 (5.6%)	0.233
Post Nasal Discharge	6 (3.4%)	5 (5.6%)	**0.048**
Thick Nasal Discharge	4 (2.3%)	2 (2.2%)	0.075
Decreased Sense of Smell/Taste	18 (10.2%)	8 (9.0%)	0.922
**Medical Comorbidities**			0.154
0	112 (63.6%)	49 (55.1%)	
1	8 (4.5%)	9 (10.1%)	
≥ 2	56 (31.8%)	31 (34.8%)	

Note: Summary statistics shown as frequency (percentage) (n [%]) unless noted otherwise.

*SNOT subdomains were based on the following categories: 1: no problem, 2: very mild, 3: moderate, 4: fairly bad, 5: severe, 6: as bad as it can be.

**, p-values generate by Chi-square/Fisher’s Exact tests for categorical variables and Wilcoxon-Rank Sum test for continuous variables.

Significantly more individuals with altered smell were SARS-CoV-2 IgG antibody positive (71.6%) than IgG antibody negative (56.7%, p = 0.047). Similarly, more participants with altered taste were IgG positive (71.0%) than negative (56.7%, p = 0.033). Alterations in smell and taste were the only studied symptoms found to be significantly higher in the positive antibody cohort; there were no significant differences between the positive and negative antibody cohorts with respect to fever and cough, and dyspnea had a higher prevalence in the negative antibody cohort (p = 0.012). Hospitalization, duration of illness, and smoking status were also not associated with antibody titer.

### Predictive modeling

Univariable logistic models demonstrated that altered smell and male sex were significant predictors of binary IgG response to SARS-CoV-2 (positive/negative, Table 3). The odds of developing IgG antibodies were 1.98 times higher among those with altered smell compared to those with normal smell (95% CI 1.14–3.42, p = 0.014). Multivariable logistic models demonstrated that male sex remained a significant predictor of positive IgG response (OR = 2.84, 95% CI 1.56–5.40, p = 0.001) when adjusting for smell. Altered smell remained a significant predictor of positive IgG response (OR = 1.90, 95% CI 1.05–3.44, p = 0.033) when adjusting for sex, age, race/ethnicity, symptom duration, hospitalization, presence of fever, smoking status, and comorbidities index. The area under the ROC curve (AUC) was 0.65 (0.58, 0.71) and the area under the precision-recall curve (AUPRC) was 0.76 (95% CI 0.68–0.83) indicating good discrimination ability of the model to distinguish between positive and negative IgG responses (S2 Table).

**Table 3:** Univariable and multivariable logistic models using SMELL as the main predictor.

	Univariable Model				Multivariable Model			
	Coeff (SE)	OR (95% CI)	p.value	Overall p.value	Coeff (SE)	OR (95% CI)	p.value	Overall p.value
Age (years)								
18–30	ref	ref	.	0.683	.	.	.	.
31–50	-0.27 (0.35)	0.77 (0.38, 1.49)	0.442	.	.	.	.	.
51+	-0.08 (0.40)	0.93 (0.42, 2.02)	0.849	.	.	.	.	.
Race								
Other	ref	ref	.	.	.	.	.	.
White (Non-Hispanic)	-0.39 (0.35)	0.68 (0.33, 1.33)	0.277	0.277	.	.	.	.
Sex								
Female	ref	ref	.	.	ref	ref	.	.
Male	1.00 (0.30)	2.71 (1.53, 4.98)	0.001	**0.001**	1.04 (0.32)	2.84 (1.56, 5.40)	0.001	**0.001**
Symptom duration (days)								
0–3	ref							
		ref	.	0.592	.	.	.	.
4–7	0.75 (0.56)	2.12 (0.70, 6.48)	0.179	.	.	.	.	.
8–11	0.82 (0.55)	2.28 (0.77, 6.80)	0.134	.	.	.	.	.
12–15	0.58 (0.53)	1.79 (0.63, 5.09)	0.271	.	.	.	.	.
>15	0.82 (0.55)	2.28 (0.77, 6.80)	0.134	.	.	.	.	.
Hospitalized								
No	ref	ref	.	.	.	.	.	.
Yes	0.44 (1.16)	1.55 (0.20,31.64)	0.705	0.705	.	.	.	.
Fever								
No	ref	ref	.	.	.	.	.	.
Yes	0.06 (0.27)	1.07 (0.63, 1.80)	0.809	.	.	.	.	.
Smoking								
Non-smoker	ref	ref	.	0.783	.	.	.	.
Former smoker	-0.27 (0.45)	0.76 (0.32, 1.90)	0.547	.	.	.	.	.
Current smoker	0.39 (1.16)	1.47 (0.19,30.01)	0.740	.	.	.	.	.
Comorbidities index	-0.09 (0.06)	0.92 (0.81, 1.04)	0.170	.	.	.	.	.
Smell								
Normal	ref	ref	.	**0.050**	ref	ref	.	**0.103**
Altered	0.68 (0.28)	1.98 (1.14, 3.42)	**0.014**	.	0.64 (0.30)	1.90 (1.05, 3.44)	**0.033**	.
Unanswered/Others	0.29 (0.76)	1.33 (0.31, 6.85)	0.706	.	0.49 (0.78)	1.62 (0.36, 8.59)	0.535	.

Multivariate modeling using IgG threshold seropositivity. Coeff (SE): regression coefficient and standard error, OR (95% CI): odds ratio and 95% confidence interval.

Separate univariable logistic models were also developed using taste as a predictor of binary IgG response (Table 4). The odds of developing IgG antibodies were 2.02 times higher among those with altered taste compared to those with normal taste (95% CI 1.17–3.48, p = 0.011). Multivariable logistic models demonstrated that male sex remained a significant predictor of positive IgG response (OR = 2.84, 95% CI 1.55–5.38, p = 0.001) when adjusting for taste, age, race/ethnicity, symptom duration, hospitalization, presence of fever, smoking status, and comorbidities index. In the same multivariable model, altered taste remained a significant predictor of positive IgG response (OR = 2.01, 95% CI = 1.12–3.61, p = 0.019) when adjusting for the other covariates. This model had an AUC of 0.65 (95% CI 0.58, 0.72) and AUPRC of 0.76 (95% CI 0.69–0.83) indicating similar discrimination ability to the smell model (S1 Table).

**Table 4:** Univariable and multivariable logistic models using TASTE as the main predictor.

	Univariable Model				Multivariable Model			
	Coeff (SE)	OR (95% CI)	p.value	Overall p.value	Coeff (SE)	OR (95% CI)	p.value	Overall p.value
Age (years)							.	.
18–30	ref	ref	.	0.683	.	.		
31–50	-0.27 (0.35)	0.77 (0.38, 1.49)	0.442	.	.	.	.	.
51+	-0.08 (0.40)	0.93 (0.42, 2.02)	0.849	.	.	.	.	.
Race							.	.
Other	ref	ref	.	.	.	.		
White (Non-Hispanic)	-0.39 (0.35)	0.68 (0.33, 1.33)	0.277	0.277	.	.	.	.
Sex							.	.
Female	ref	ref	.	.	ref	ref		
Male	1.00 (0.30)	2.71 (1.53, 4.98)	0.001	**0.001**	1.04 (0.32)	2.84 (1.55, 5.38)	0.001	**0.001**
Symptom duration (days)							.	.
0–3	ref	ref	.	0.592	.	.		
4–7	0.75 (0.56)	2.12 (0.70, 6.48)	0.179	.	.	.	.	.
8–11	0.82 (0.55)	2.28 (0.77, 6.80)	0.134	.	.	.	.	.
12–15	0.58 (0.53)	1.79 (0.63, 5.09)	0.271	.	.	.	.	.
>15	0.82 (0.55)	2.28 (0.77, 6.80)	0.134	.	.	.	.	.
Hospitalized							.	.
No	ref	ref	.	.	.	.		
Yes	0.44 (1.16)	1.55 (0.20,31.64)	0.705	0.705	.	.	.	.
Fever							.	.
No	ref	ref	.	.	.	.		
Yes	0.06 (0.27)	1.07 (0.63, 1.80)	0.809	.	.	.	.	.
Smoking							.	.
Non-smoker	ref	ref	.	0.783	.	.		
Former smoker	-0.27 (0.45)	0.76 (0.32, 1.90)	0.547	.	.	.	.	.
Current smoker	0.39 (1.16)	1.47 (0.19,30.01)	0.740	.	.	.	.	.
Comorbidities index	-0.09 (0.06)	0.92 (0.81, 1.04)	0.170	.	.	.		
Taste							.	**0.056**
Normal	ref	ref	.	**0.036**	ref	ref		
Altered	0.70 (0.28)	2.02 (1.17, 3.48)	**0.011**	.	0.70 (0.30)	2.01 (1.12, 3.61)	**0.019**	.
Unanswered/Others	0.90 (0.85)	2.47 (0.53, 17.52)	0.286	.	0.92 (0.88)	2.52 (0.49, 18.68)	0.295	.

Coeff (SE): regression coefficient and standard error, OR (95% CI): odds ratio and 95% confidence interval.

## Discussion

This study demonstrates that altered smell and taste are associated with a robust serologic response to SARS-CoV-2. The association remains significant when adjusting for other predictors of serologic response, including age and sex. These findings have important implications as we continue to combat the ongoing pandemic. Although vaccination efforts against COVID-19 have been successful in parts of the world [31], nearly half of the world’s population remains unvaccinated [32]. Overall, examining factors associated with a serologic response to SARS-CoV-2 is an important step toward understanding the pathophysiology of this disease, identifying mechanisms of natural immunity, and developing targeted vaccines and global vaccination strategies.

Other studies have sought to identify factors associated with antibody response to SARS-CoV-2. Among these, disease severity remains the most studied and contested association. Although time to seroconversion appears independent of disease severity [33], elevated and persistent levels of IgG are characteristic of severe disease [34]. There may be an association between mild and asymptomatic SARS-CoV-2 infection and the robustness of immunologic response; however, some reports have argued against this association. For example, one study on the kinetics of SARS-CoV-2 IgM and IgG responses in ICU vs non-ICU patients (with ICU admission serving as a proxy for higher disease severity) found that IgG against the spike protein was higher in non-ICU patients than ICU patients by 2 weeks after disease onset [35].

Loss of both smell and taste have been universally recognized as key symptoms of SARS-CoV-2 infection. Prior reports demonstrate an opportunity to leverage smell and taste alteration to predict diagnosis of COVID-19 [1, 36, 37]. However, the capacity for chemosensory deficits to predict immunologic response has not been previously explored. Our results suggest the presence of a robust anti-Spike IgG response in individuals experiencing smell and taste loss during COVID-19 infection. Heterogeneity in accuracy of testing methods, varied assessment of IgA, IgM, and IgG, and classification of seropositivity in a binary fashion contribute to the challenge in consolidating findings from serologic studies.

The current study builds upon a growing body of literature on immunity to SARS-CoV-2. Interestingly, our results demonstrate that IgG positivity was associated not only with chemosensory dysfunction, but also male sex. This is consistent with a previous report that identified that men develop higher levels of IgG antibodies against the S1 viral subunit of SARS-CoV-2 spike and neutralizing antibodies compared to women [38]. In the current study, however, markers of COVID-19 severity, including duration of symptoms and whether hospitalization was required, were not predictive of IgG positivity. Moreover, dyspnea, often considered a characteristic of more severe disease, was associated with negative IgG status. Although this finding may reflect a heterogeneity in dyspnea severity not captured in our study, these results suggest that additional research is necessary to elucidate the relationship between disease severity and seropositivity.

This study has several limitations, including cross-sectional design that prevents characterization of the durability of immune response over time, the convenience sample nature of the participant population that is restricted to those able to participate in CPT, the lack of direct neutralization assays, as antibody levels do not confer immunity [39], and the ongoing evolution of SARS-CoV-2 resulting in variant-specific behavior. This study also does not examine anti-nucleoprotein (anti-N) IgG response, which may also be related to disease severity [35]. Future research should examine the prevalence of chemosensory dysfunction and seropositivity among individuals following vaccination, those infected with distinct SARS-CoV-2 variants/sub-variants, and those with subsequent exposures and development of COVID-19 illness.

It is important to note that the accuracy of our results may also be limited by the self-reported and symptom-based diagnosis of smell and taste alteration during SARS-CoV-2 infection. At the time of data collection, restrictions on SARS-CoV-2 testing availability and ongoing efforts to reduce social contact prevented integration of objective psychophysical testing to assess olfactory thresholds. Given the subjective nature of these variables, participants may have struggled to recall whether their sense of smell or taste remained intact or was altered. It may also be challenging to notice chemosensory dysfunction in the presence of more concerning symptoms during acute infection, such as respiratory distress. These factors may have led to underreporting of symptoms. Despite these limitations, previous studies have demonstrated a strong correlation between self-reported smell loss during the pandemic and objective assessment of smell loss [6, 10]. Taste alteration can be more difficult to measure and its objective prevalence in COVID-19 has been debated more than that of smell. Without objective testing, it is difficult to parse out whether a patient reporting taste disturbance may have an underlying issue solely attributable to smell dysfunction. However, given the close relationship between smell and taste perception, particularly as experienced and reported by the general public throughout the course of the pandemic with taste loss often endorsed even in the absence of reported smell loss, both variables were included in the current study to provide a comprehensive assessment of chemosensory function in each patient.

## Conclusion

Understanding prevalence and duration of immunologic response among patients with history of SARS-CoV-2 infection and individuals undergoing immunization remains a priority as we look toward societal emergence from the COVID-19 pandemic. Results from our study suggest that loss of smell and taste during COVID-19 infection are strong predictive factors for a robust immunologic response based on IgG titers. Additional research is needed to address the durability of seropositivity among these individuals.

## Supporting information

S1 Table Sample survey questions evaluating olfactory function (same questions asked for gustatory function). (DOCX)

S2 Table Multivariable models performance assessment. (DOCX)

S1 Data (XLSX)
