# Supplementary material for: Chemosensory deficits are best predictor of serologic response among individuals infected with SARS-CoV-2
Source: PLoS One. 2022 Dec 14;17(12):e0274611. doi: 10.1371/journal.pone.0274611 (PMC9750016; doi:10.1371/journal.pone.0274611)
Supplement: S1 Table — (DOCX) [file pone.0274611.s001.docx]

**Table S1. Sample survey questions evaluating olfactory function (same questions asked for gustatory function).**

| **Prior to the onset of coronavirus symptoms, your baseline awareness of SMELL was:** | - Very unaware (I can't smell). - Unaware, (Most friends are able to smell more). - Normal, (my sense of smell is similar to friends). - Aware, (my sense of smell is sensitive). - Very aware, (my sense of smell is exquisitely sensitive). |
| --- | --- |
| **In the 2 weeks during your coronavirus symptoms your SMELL was (select all that apply):** | - Normal, my baseline/usual. - Absent, my sense of smell was completely gone. - Diminished, my sense of smell was less than normal. - Heightened, my sense of smell was overly sensitive - to some or all scents. - Distorted, my perception of smells was different - from normal. - Odd, I smelled scents or odors that no one else - could. |
| **Currently, how do you BEST describe your sense of SMELL?** | - Normal, my baseline/usual. - Absent, my sense of smell is completely gone. - Diminished, my sense of smell is less than normal. |
| **Currently, regarding your SMELL, do you notice any of the following?** | - Heightened, my sense of smell is overly sensitive to some or all scents. - Distorted, my perception of smells is different from normal. - Odd, I smell scents or odors that no one else can. |
| **How often do you notice your SMELL problem?** | - All the time. - Some of the time, comes and goes. |
