# Supplementary material for: Chemosensory deficits are best predictor of serologic response among individuals infected with SARS-CoV-2
Source: PLoS One. 2022 Dec 14;17(12):e0274611. doi: 10.1371/journal.pone.0274611 (PMC9750016; doi:10.1371/journal.pone.0274611)
Supplement: S2 Table — (DOCX) [file pone.0274611.s002.docx]

**Table S2. Multivariable Models Performance Assessment**

|  | **Smell Model (Table 3)** | **Taste Model (Table 4)** |
| --- | --- | --- |
| **AUC** | 0.65 (0.58, 0.71) | 0.65 (0.58, 0.72) |
| **AUPRC** | 0.76 (0.68, 0.83) | 0.76 (0.69, 0.83) |
| **PPV** | 0.71 (0.64, 0.77) | 0.72 (0.65, 0.78) |
| **Calibration Slope** | 1.02 (0.47, 1.63) | 1.02 (0.51, 1.60) |

AUC (area under the ROC curve); AUPRC (area under the precision-recall curve); PPV (positive predictive value)

Calibration slope (closer to 1 indicates good calibration): slope < 1 indicates overfitting, slope > 1 indicates underfitting.
